# An Efficient Local Molecular Dynamics Polymerization Simulation Combined with an *Ab Initio* MO Method

**DOI:** 10.3390/ma6030870

**Published:** 2013-03-06

**Authors:** Peng Xie, Yuuichi Orimoto, Yuriko Aoki

**Affiliations:** 1 Department of Molecular and Material Sciences, Interdisciplinary Graduate School of Engineering Sciences, Kyushu University, 6-1 Kasuga-Park, Fukuoka 816-8580, Japan; E-Mail: ak-x-peng@mms.kyushu-u.ac.jp; 2 Department of Material Sciences, Faculty of Engineering Sciences, Kyushu University, 6-1 Kasuga-Park, Fukuoka 816-8580, Japan; E-Mail: orimoto.yuuichi.727@m.kyushu-u.ac.jp; 3 Japan Science and Technology Agency, CREST, 4-1-8 Hon-chou, Kawaguchi, Saitama 332-0012, Japan

**Keywords:** elongation method, molecular dynamics, helix forming, hydrogen bond

## Abstract

A new local *ab initio* molecular dynamics method, namely elongation molecular dynamics (ELG-MD) is proposed for highly efficient simulations of aperiodic polymer systems. ELG-MD combines the elongation method (ELG) with the Gear predictor corrector (GPC) algorithm of molecular dynamics simulation. In this method, the local gradients acting on the atom’s nucleus in the active region are calculated by the ELG method while the equations of the nucleus’s motion are solved by the GPC algorithm. In this work, the first application of this ELG-MD method is described to investigate the stable conformation of polyglycine with surrounding water molecules. The water effects on the structure of polyglycine are examined. The ELG-MD simulations show that the formation of the polyglycine helix is strongly induced by the hydrogen bonds observed in two types of H-bond rings.

## 1. Introduction

Computer simulations on biological macromolecules have been a hot area for numerous theoretical studies describing biological processes at the molecular level. One of the popular treatments for this purpose is the classical molecular dynamics simulation method in which empirical interatomic potential functions are used. However, it is not so accurate because of the treatment of constant atom charges. On the other hand, in *ab initio* molecular dynamics (AIMD), the electronic structure, energy and forces of the system are directly computed based on quantum mechanics theory. However, the number of atoms one can handle in the quantum mechanical treatment is much smaller than the number of atoms in a biological system because of the limitation of computer resources. The elongation (ELG) method has proved to be an efficient method for calculating the electronic structure of large aperiodic polymers with high accuracy at the *ab initio* level [1,2,3,4,5,6]. In addition, the analytical energy gradients can be calculated by the ELG method and have been successfully used for the geometry optimization of a series of polymers [7]. Based on these developments, we here propose a new efficient AIMD method named Elongation-MD (ELG-MD) by combining the elongation method with the dynamics algorithm. The ELG-MD method makes it possible to efficiently analyze the mechanisms of chemical reactions by considering dynamics even for huge random polymers such as biomaterials. For the first application of the ELG-MD method to confirm its effectiveness, we selected polyglycine as a target system; polyglycine is representative of the simplest peptide and widely investigated both experimentally and theoretically.

Polyglycine (Gly)*_n_*, containing n residues, forms the backbones of amino acids, peptides and proteins without side chains and their functional groups [8]. Therefore, the investigations on (Gly)*_n_* are important to the understanding of a wide range of biological systems. In particular, the three-dimensional structure is one key factor for the biological activities of a peptide, and hydrogen bonds play important roles in stabilizing the secondary structure of peptides [9]. In general, hydrogen bonds (H-bonds) can be generated both intramolecularly and intermolecularly in the system. In the former case, the H-bonds are formed between many different functional groups in the peptide, but in the latter case the H-bonds are formed between polar groups of the peptide and the surrounding solvent molecules such as water molecules. For a β strand, the hydrogen bonds are formed by the N–H groups in the backbone of one β strand with the C=O groups in the backbone of the adjacent β strands. For an α helix, each hydrogen bond is formed by the N–H group of an amino acid with the C=O group of the amino acid four residues earlier. On the conformation of polyglycine, Ohnishi *et al.* demonstrated that polyglycine in solution exhibits a strong preference for an extended conformation when polymerized in short segments as the intermediate in the extension between a β strand and an α helix [10]. Which kind of hydrogen bond will stabilize this extended conformation of polyglycine in aqueous solution? The hydration effects via direct hydrogen bonds between water molecules and the peptide backbone can be expected. Here we applied the ELG-MD method to investigate the intermolecular H-bonds effects on conformation stability when (Gly)*_n_* is polymerized with water molecules. This application deals with the conformation change of (Gly)*_n_* under the explicit consideration of water molecules, and the AIMD simulation in which energies and forces are generated by the ELG method at each time step.

This paper is organized into four sections. In Section 2, we briefly introduce the ELG formalism including energy and gradient calculations, after that we present a description of the ELG-MD method. In Section 3, we introduce the structure of model molecules, *i.e.*, polyglycine with water molecules. In the same section we analyze the equilibrium structures of polyglycine and their energetic stability. Final conclusions are collected in Section 4.

## 2. Methodology

### 2.1. Elongation Method

**Figure 1:**
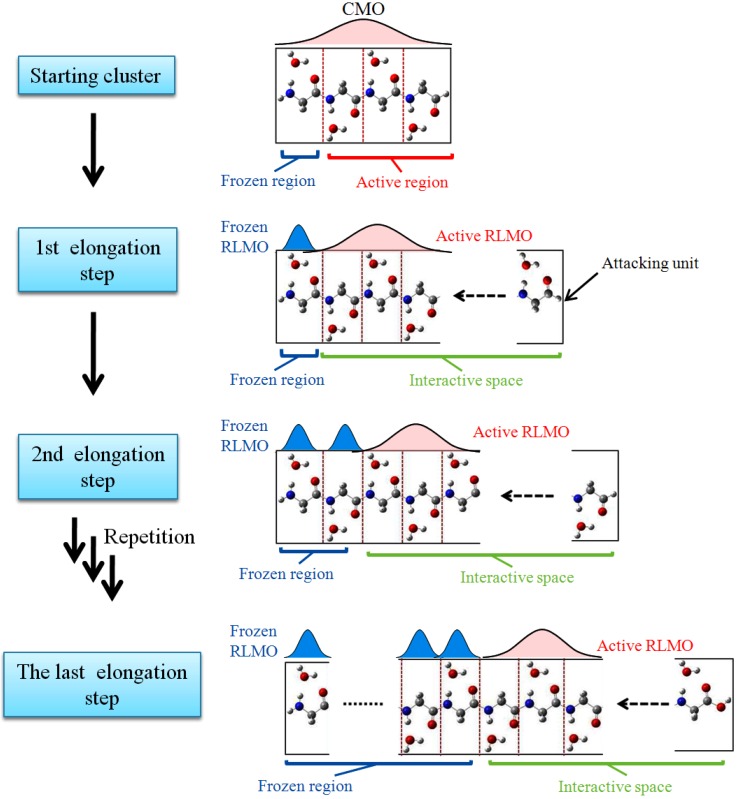
Flowchart of the elongation method illustrated using polyglycine with water molecules. The canonical molecular orbital (CMO) and region localized molecular orbital (RLMO) indicate canonical and regional localized molecular orbitals, respectively.

The elongation method can be considered as a procedure for *theoretical* polymerizations on computers [1,2,3]. We present a brief overview of the procedures (see also the flowchart in Figure 1).

First, the polymer chain is divided into several monomers, and then a suitable size of monomers as a starting cluster is selected. A and B regions are defined for the starting cluster, where the A region is the frozen region assumed to be the side furthest away from the chain propagation point, and the B region is the active region near the propagation point. The canonical molecular orbitals (CMOs) of the starting cluster are solved by the conventional Hartree-Fock self-consistent-field (HF-SCF) procedure. 

Second, the CMOs are converted to localized MOs (LMOs) over the A or B regions. We define the interacting space as the B region with an attacking molecule, and the A region is furthest away from the interaction spot. The localization procedure is explained in the reference [2]. The obtained LMOs are localized in a special region over several units, and are different from those by Ruedenberg’s [11] or Boys’s [12] localization method that provide LMOs localized on a bond. Thus, we name the obtained orbitals as region localized molecular orbitals (RLMOs). By using the RLMOs, the density matrix can be expressed by

(1) $$D^{\text{RLMO}}=\left\{L_{\text{CMO}}^{\text{RLMO}}(A)+L_{\text{CMO}}^{\text{RLMO}}(B)\right\}P\left\{L_{\text{RLMO}}^{\text{CMO}}(A)+L_{\text{RLMO}}^{\text{CMO}}(B)\right\}$$

where *L* indicates the transformation matrix between CMO and RLMO. *P* is the diagonalized matrix in which the degenerates 2 and 0 are given as the eigenvalues for occupied and vacant spaces, respectively. The *D*^RLMO^ is actually identical to *P* because the transformation matrix *L* is defined under the freedom that comes from the non-uniqueness of the coefficients owing to the degeneracy within all the occupied orbitals and all the vacant orbitals. In other words, the occupied and vacant spaces are not mixed with each other by Equation (1). Therefore, the diagonalized density matrix *P* is invariant under the unitary transformation. By this feature, we can make preferable RLMOs suitable for local interaction during the polymerization process.

Third, at the elongation step, the attacking monomer (M) is attached to the terminal of the B region. Because the A region is far away from the M region, the interaction between them is negligible. Thus, the ELG HF equation is solved self-consistently only for the localized orbital space including the B and M regions. After the new CMOs of the B and M regions are obtained, the new CMOs are localized into new frozen (A′) and active (B′) regions. Then a new attacking monomer (M’) is attached. The above procedures are repeated step by step until the electronic structure of the target system is obtained.

### 2.2. Elongation Gradient Calculation

In HF approximation, the total energy of a closed-shell system can be written as

(2) $$E=\sum_{\mu \;\nu }P_{\nu \;\mu }H_{\mu \nu }^{\text{core}}+\frac{1}{2}\sum_{\mu \nu \lambda \sigma }P_{\mu \;\nu }P_{\lambda \;\sigma }(\mu \nu ∥\sigma \lambda )+V_{\text{NN}}$$

where *P*, *H*^core^ and *S* denote the density matrix, core Hamiltonian and overlap matrix of the system, respectively. The (*μν*||*σλ*) and *V*_NN_ represent the two-electron integral and the nuclear-nuclear repulsion, respectively. We can obtain the first derivative (gradient) of the total energy *E* by the analytic derivative method with the form of explicit dependence on the nuclear coordinate *X*_A_ [13] as

(3) $$\frac{\partial E}{\partial X_{A}}=\sum_{\mu \nu }P_{\nu \;\mu }\frac{\partial H_{\mu \nu }^{\text{core}}}{\partial X_{A}}+\frac{1}{2}\sum_{\mu \nu \lambda \sigma }P_{\nu \;\mu }P_{\lambda \;\sigma }\frac{\partial (\mu \nu ∥\lambda \sigma )}{\partial X_{A}}-\sum_{\mu \nu }Q_{\nu \;\mu }\frac{\partial S_{\mu \nu }}{\partial X_{A}}+\frac{\partial V_{\text{NN}}}{\partial X_{A}}$$

where Q is the energy-weighted density matrix defined as

(4) $$Q_{\nu \;\mu }=2\sum_{i}^{N/2}\varepsilon_{i}C_{\mu \;i}C_{\nu \;i}$$

A detailed description of how to obtain gradient calculations with the elongation method was presented in a recent paper of our group [7]. We sketch this method out here. Because the elongation method is based on AO basis, the density matrix by the ELG method is almost the same as the density matrix in Equation (3) for the conventional calculation except for the third term *Q_νμ_*. In each elongation step, the HF equations are solved only for the interacting space including the B and M regions. After the ELG-SCF procedure, the molecular orbitals of the B and M regions are transformed from RLMO to AO basis for calculating the gradient in Equation (3). First, we combine $C_{\text{A}}^{\mathrm{RLMO}}$ with $C_{\text{BM}}^{\text{MO}}$ to construct the coefficient matrix for the whole system, *C*^MO^, where the $C_{A}^{\text{RLMO}}$ and $C_{\text{BM}}^{\text{MO}}$ denote the coefficients of the frozen region (A) from the previous ELG steps and interacting space (B + M) from the ELG-SCF calculation in the present ELG step, respectively. The *ε* matrix can be calculated by *C*^MO^*FC*^MO^, but the *ε* is not diagonal any more. Therefore, we can get the AO based energy-weighted density matrix Q for the ELG-HF method by

(5) $$Q_{\nu \;\mu }=\sum_{i\;j}^{N/2}n_{i}\varepsilon_{i\;j}C_{\mu \;i}C_{\nu \;j}$$

where *n* is the occupancy number matrix corresponding to the density matrix in the MO representation, in this matrix degenerate 2 is given for the occupied space and degenerate 0 is for the vacant space.

### 2.3. Elongation Molecular Dynamics

We can straightforwardly combine the ELG method with the MD algorithm to construct the ELG-MD method in the following manner. Newton’s equation of motion is solved by calculating the ELG gradients on the fly using

(6) $$F=m\frac{d^{2}r}{d\;t^{2}}=-\nabla \;E^{\text{ELG}}$$

The time evolution of the atomic nuclei is performed according to the fourth order Gear predictor corrector (GPC) algorithm [14,15]. The GPC is one of the most widely-used algorithms in molecular dynamics simulations. In general, any time-dependent property can be estimated from a Taylor series expansion. In the predictor stage of the GPC, therefore, the atomic coordinate vector *r^p^* can be expanded in the Taylor series in the time step Δ*t*:

(7) $$r^{p}(t+\Delta t)=r(t)+\frac{dr}{dt}\Delta t+\frac{1}{2}\frac{d^{2}r}{dt^{2}}\Delta t^{2}+\frac{1}{6}\frac{d^{3}r}{dt^{3}}\Delta t^{3}+\frac{1}{24}\frac{d^{4}r}{dt^{4}}\Delta t^{4}+\ldots$$

The atomic velocity vector *v^p^* and the acceleration vector *a^p^* could be updated by the other appropriate expansions similar to Equation (7), respectively. We here define the first, second, and third derivative of *r* as *v* = $\frac{dr}{dt},$ *a* = $\frac{d^{2}r}{dt^{2}}$ and *b* = $\frac{d^{3}r}{dt^{3}},$ respectively.

(8) $$v^{p}(t+\Delta t)=v(t)+\frac{d^{2}r}{dt^{2}}\Delta t+\frac{1}{2}\frac{d^{3}r}{dt^{3}}\Delta t^{2}+\ldots$$

(9) $$a^{p}(t+\Delta t)=a(t)+\frac{d^{3}r}{dt^{3}}\Delta t+\ldots$$

(10) $$b^{p}(t+\Delta t)=b(t)+\ldots$$

Although we get a set of predicted vectors from these procedures, they are not correct because of their truncation errors. Thus, all the vectors are corrected in the corrector stage of GPC. From the new predicted coordinates, we can actually calculate the accelerations *a^c^*. The difference between *a^c^* and *a^p^*, *i.e.*, Δ*a*, are used to correct the predicted value.

(11) $$\Delta a(t+\Delta t)=a^{c}(t+\Delta t)-a^{p}(t+\Delta t)$$

(12) $$r^{c}(t+\Delta t)=r^{p}(t)+c_{0}\Delta a(t+\Delta t)$$

(13) $$v^{c}(t+\Delta t)=v^{p}(t)+c_{1}\Delta a(t+\Delta t)$$

For example, suitable values of *c*_0_ and *c*_1_ for the fourth-order GPC algorithm are 19/120 and 3/4, respectively. In a similar way, the other vectors can be also corrected. 

**Figure 2:**
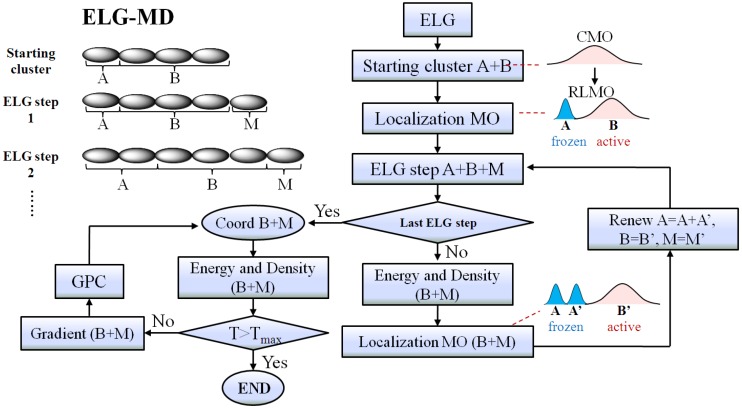
The flowchart of the elongation molecular dynamics (ELG-MD) method. The “Energy and Density (B + M)” means that the energy and density of the B + M region are obtained with the contribution from the A region.

As mentioned above, the ELG method is similar to the procedure for the theoretical synthesis of polymers. When the attacking monomer (M region) approaches the B region, the structure of the interacting space (B and M regions) will change because of the interaction between them. In contrast, the frozen region (A region) is far away from the M region and thus we can presume there is no interaction between the A region and the M region. Therefore, the structure of the A region will not change any more when M approaches B. For this reason, in the ELG-MD calculation, we only perform molecular dynamics on the interacting space in the last step of ELG-MD. For the previous ELG steps, only energy calculations are carried out. The flowchart of the ELG-MD method is presented in Figure 2. On the right side of Figure 2, it is shown that the A, B and M regions are renewed for the next elongation step after localization, so A denotes a different region in a different elongation step, as do B and M. All the procedures of the ELG-MD method were implemented with the GAMESS program package [16].

## 3. Results and discussion

**Figure 3:**
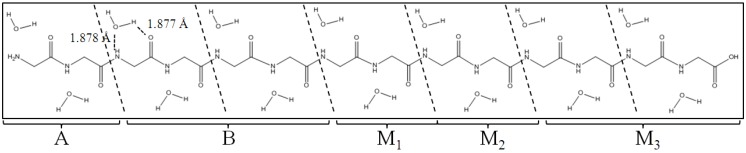
Polyglycine (Gly)_14_ in β-strand conformation with 14 water molecules. (Gly)_14_ are divided into A, B, M_1_, M_2_ and M_3_ regions for the ELG-MD procedures. The notations A and B do not correspond to those in Figure 2.

**Figure 4:**
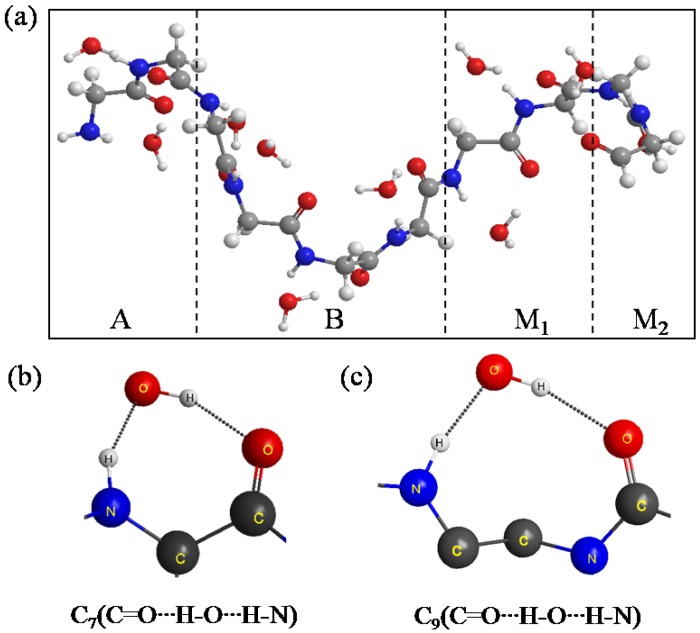
(**a**) The structure of the A, B, M_1_ and M_2_ regions of (Gly)_14_ in a quasi-α-helix conformation as the initial structure for ELG-MD simulation. Panels (**b**) and (**c**) show two types of H-bond ring. The dotted lines in the panels denote hydrogen bonds.

A (Gly)_14_ peptide in β-strand conformation with 14 water molecules was selected as a model system to test and demonstrate the ELG-MD method (Figure 3). For the first testing calculation for ELG-MD, we did not consider more water molecules around polyglycine. One reason was to save computing time, another reason is that for the central fragment of the glycine molecule, one side including C=O and N–H groups is hydrophilic and these two groups can form a hydrogen bond ring C_7_(C=O···H–O···H–N) (shown in Figure 4b) with one water molecule, it is not easy to add more water molecules forming hydrogen bonds with these two groups in this area because of space limitation. Another side including the CH_2_ group is hydrophobic. Here, the notation C*_n_* (X···H–O···Y) represents the hydrogen bond ring structure consisting of n atoms enclosed by the H–O group of the adjacent water molecule. The (Gly)_14_ is divided into seven units ((Gly)_2_ for each unit), and assigned A, B, and M_1_~M_3_ regions as shown in Figure 3. The simulation in the present article has two stages.

First, we need a stable conformation of A, B, M_1_, and M_2_ regions to create a more real surrounding for the new attacking monomer (M_3_ region) before investigating the structure of (Gly)_14_ including the A, B, and M_1_~M_3_ regions. Several research groups have devised experiments for measuring β-sheet stability with various peptides, and they found polyglycine has a low β-sheet propensity [17,18,19,20]. Therefore, to find a more reasonable conformation than β-sheet conformation, the conventional AIMD simulation was performed for the A, B, M_1_ and M_2_ regions (other than the M_3_ region). In the AIMD simulation, the energies and forces were obtained at the HF level of theory with the STO-3G basis set. The time step is set as 0.5 fs and we perform in total 5 ps simulation (10,000 steps). The temperature of the system is associated with the classical kinetic energy and the constant temperature (298 K) is achieved by the algorithm proposed by Berendsen *et al.* [21]. Figure 4a shows the equilibrium structure obtained by the simulation. It was found that the conformation of A, B, M_1_ and M_2_ significantly changed from a β-sheet type to a quasi-α-helix type in which the structure is more outstretched than α-helix. In general, α-helix forms a right-handed helix and every N–H group in the backbone donates a hydrogen bond to the C=O group of the amino acid belonging to four residues earlier (I + 4 → i hydrogen bonding) [22]. Our results in Figure 4a show that the right-handed helix is induced by two kind of hydrogen bond rings, that is, C_7_(C=O···H–O···H–N) in Figure 4b and C_9_(C=O···H–O···H–N) in Figure 4c. C_9_(C=O···H–O···H–N) shows that the H–O group of the water donates two hydrogen bonds to the C=O and N–H groups of the backbone for instance. 

**Figure 5:**
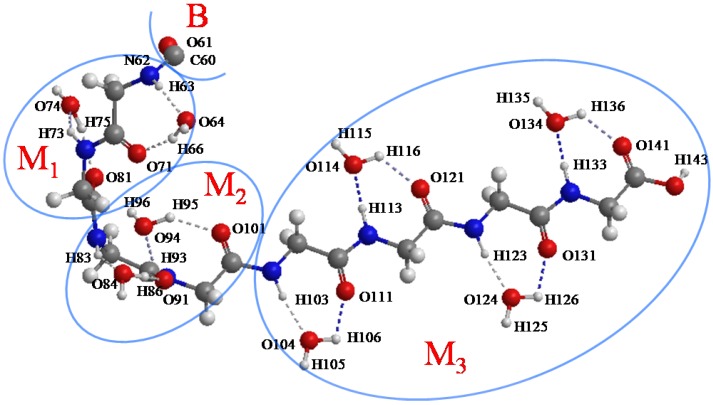
The initial structure of the M_1_, M_2_ and M_3_ regions (atoms from 62 to 143). C60 and O61 belong to the B region.

Second, for the ELG-MD calculation, there are four calculation steps including starting cluster (A and B regions), the first elongation step (A, B and M_1_ regions), the second elongation step (A, B, M_1_ and M_2_ regions) the third elongation step (A, B, M_1_, M_2_ and M_3_ regions). Energy calculations are carried out for the first three steps, and MD only performed for the last step. In the last step, we presume that a new monomer M_3_ (β form) furthermore attacks the propagating site of the M_2_ region of the structure obtained above. Therefore, the initial structure for the ELG-MD method consists of the A + B + M_1_ + M_2_ equilibrium structure (quasi-α form) and an additional M_3_ region (β form). The M_1_~M_3_ region of the initial structure is shown in Figure 5. Because we are interested in the structure variation of M_3_ and the associated regions such as the M_1_, M_2_ regions, the MD treatment in the ELG-MD procedure is applied only for the interacting space in the last elongation step, that is, the M_1_~M_3_ region while the A and B regions are frozen. For the dynamics part of the ELG-MD calculation, we used the same setting as conventional AIMD simulation. 

**Figure 6:**
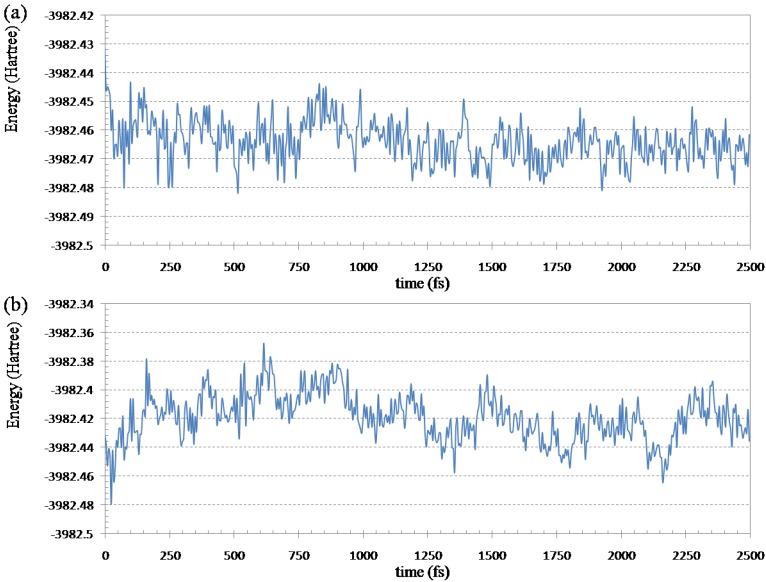
Fluctuations of the Hartree-Fock energy of (Gly)_14_ with 14 water molecules in the simulation at 298.15 K: (**a**) ELG-MD simulation; (**b**) conventional *ab initio* MD simulation.

To know the efficiency of the ELG-MD method, 2.5 ps (5000 steps) simulation was performed by both ELG-MD and conventional AIMD for the initial structure in Figure 5. The total wall clock time is 206,362.3 s (ELG-MD) and 298,391.3 s (conventional AIMD), respectively. Because the ELG-MD is a local MD method, its efficiency is obviously higher (about 31%) than the conventional AIMD. Furthermore, the potential energies (HF energies) of the entire system can be compared between the two treatments (see Figure 6).

From Figure 6a we can see the HF energy decreases quickly for the ELG-MD method which relaxes only a local region of the polymer. In the ELG-MD calculation, the structure of the A and B regions is automatically frozen when the M_3_ region attaches, which is different from the conventional AIMD which treats the whole system at the same time. Because the freedom of the ELG-MD calculation is much less than conventional AIMD, it is much easier for the ELG-MD method to get the energy convergence. Figure 6b shows the fluctuations of the Hartree-Fock energy calculated by conventional AIMD. The energy increases during 900 fs, and then the energy begins to decrease slowly. That is to say, it is hard to get the energy equilibrium in a short period of time in the conventional AIMD. It also should be stressed that the energy of ELG-MD is lower than that of conventional AIMD at 2500 fs. From 2000 to 2500 fs, the average total energy is −3982.4664 Hartree for ELG-MD while −3982.4245 Hartree for AIMD. When the new monomer attaches to the propagating site of the M_2_ region, it will mainly affect the structure of the M_1_ and M_2_ regions because the A and B regions are far away from the M_3_ region (the distance between them is about 10 Å). Therefore, we only focus on the conformation variation of the attacking monomer (M_3_) and its vicinity (M_1_ and M_2_) under the assumption that the A and B regions have no interactions with the M_3_ region.

**Figure 7:**
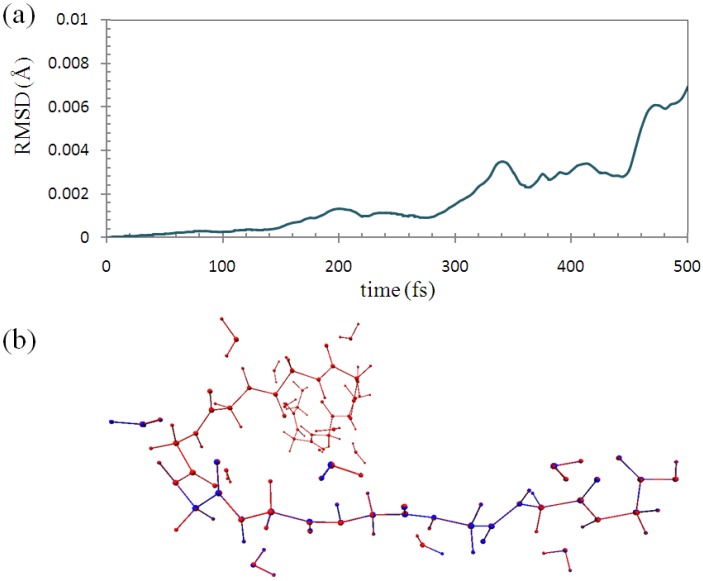
(**a**) Root-mean-square deviation (RMSD) of the molecular structure between ELG-MD and conventional *ab initio* MD with fixing of the same atoms; (**b**) Superimposed structures at 500 fs. (Red: conventional AIMD. Blue: ELG-MD).

To check the accuracy of the ELG-MD, we performed 0.5 ps (1000 steps) conventional AIMD calculation for the polymer with fixing of the atoms in the A and B regions in the same manner as with the ELG-MD calculation. We compared the structures of them in each simulation step. Figure 7a shows the root-mean-square deviation (RMSD) of the backbone part (M_1_~M_3_) of the structure between the ELG-MD and conventional AIMD with fixing the atoms. The maximum value of RMSD with 0.00695 Å is considerably small. This shows that the two structures agree well in each simulation step. 

As shown in Figure 7b, the two structures at 500 fs (1000 steps) calculated by the two methods are almost the same as each other. Hence, it can be concluded that the trajectories of the two simulations have essentially the same quality, and the structure obtained by the ELG-MD simulation is credible.

**Figure 8:**
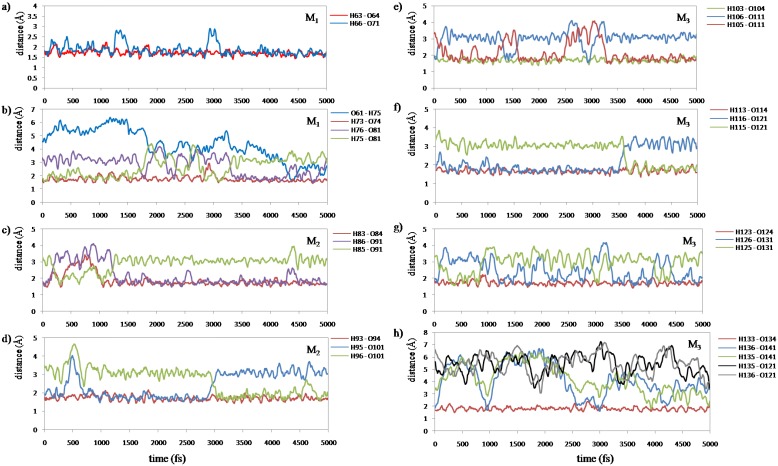
Fluctuations of the H-bond distances between peptide and water molecules in the interacting space (5 ps ELG-MD simulation). The numbering of the atoms is shown in Figure 5. M_1_, M_2_ and M_3_ denote the M_1_ region, M_2_ region and M_3_ region shown in Figure 5 respectively. More details of the H-bond distances in Panel b and h are shown in Figure 9 and Figure 10, respectively.

Now, we performed 5 ps (10,000 steps) ELG-MD simulation for the (Gly)_14_ peptide starting from the initial structure in Figure 5 to investigate the conformation variation from the viewpoint of the H-bond effects between peptide and water molecules. It should be noted that the frozen region (A and B) is not shown in Figure 5 because the coordinates of the frozen regions are fixed. As mentioned above, in the initial structure the M_3_ region has β-strand confirmation and the other region A + B + M_1_ + M_2_ has quasi-α-helix conformation. All of the H-bonds in the initial structure are of the C_7_(C=O···H–O···H–N) type. We measured all of the H-bond lengths during the simulation to examine the configuration change. We assigned these H-bonds into eight groups according to their locations. The changes in the atomic distances of these H-bonds are shown in Figure 8.

**Figure 9:**
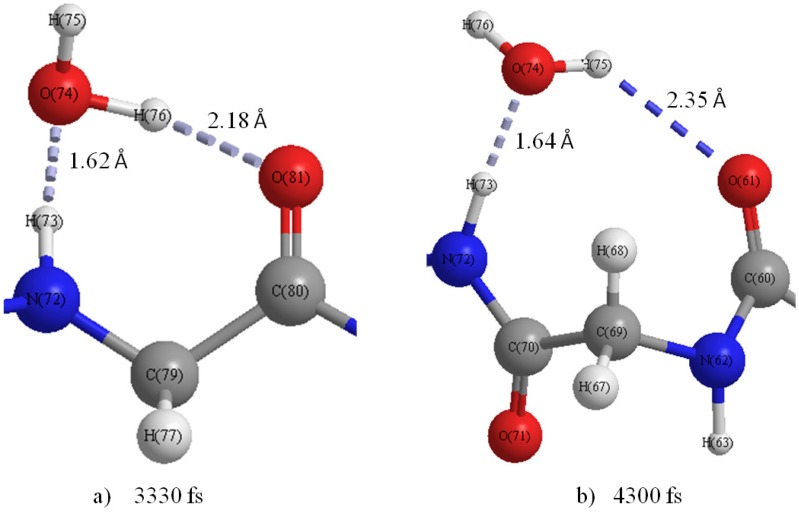
Snapshot of H-bond rings in the M_1_ region at (**a**) 3330 fs and (**b**) 4300 fs.

**Figure 10:**
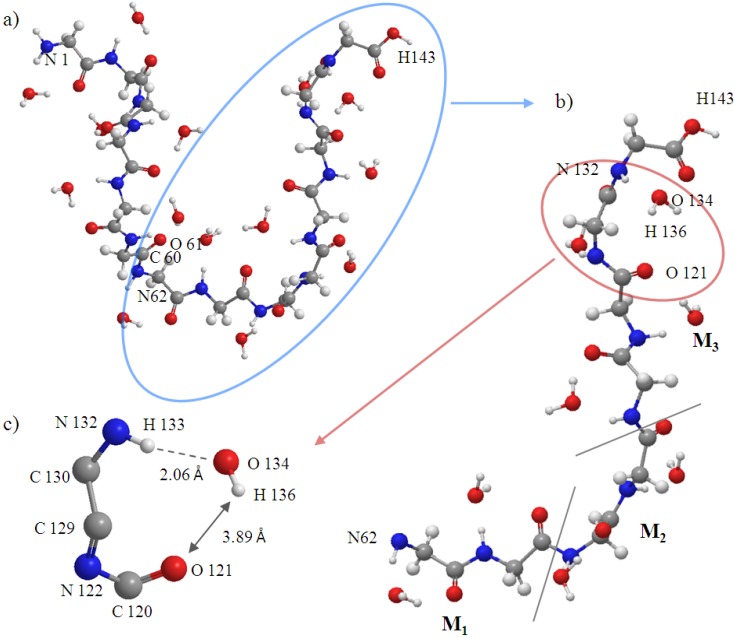
Snapshot at 5000fs of (**a**) entire peptide (Gly)_14_. Atoms from 1 to 61 belong to the frozen region; atoms from 62 to 143 belong to the active region; (**b**) Interacting space (M_1_, M_2_ and M_3_); and (**c**) H-bond.

Figure 8a shows that the H-bonds (H63···O64 and H66···O71) near the curve region in the helix are relatively stable throughout the simulation. Figure 8c–g show that the number of H-bonds is always maintained at two in these areas. In addition, the variation of H-bonds only occurs between O of the backbone’s C=O group and the two H atoms of the water molecule in the vicinity induced by the rotation of the water molecule. Figure 8b shows that the H-bond of H75···O81 changed to that of H76···O81 at 3330 fs (Figure 9a), and a new H-bond O61···H75 in C_9_(C=O···H–O···H–N) type is observed at 4300 fs (Figure 9b). Then, it was found that the H-bonds H76···O81 and O61···H75 became stable after 4300 fs. The formation of new C_9_(C=O···H–O···H–N) type H-bonds implies that the helix structure of this area becomes more stable and more compact than the initial structure. The variations of H-bonds in the tail region of the peptide are more active than the other regions as shown in Figure 8h. After 4300 fs, the H135···O141, H135···O121 and H136···O121 distances have a downward trend, but the H136···O141distance increases in contrast. This implies that the H-bond of H136···O141 gradually changes to H135···O141, and it has a tendency to form a new H-bond H136···O121 in the C_9_(C=O···H–O···H–N) type as shown in the snapshot at 5000 fs (Figure 10c). The snapshot structures of interacting space (M_1_, M_2_ and M_3_) and the entire peptide at 5000 fs are shown in Figure 10a,b, respectively. All the C_7_(C=O···H–O···H–N) type H-bonds in the peptide interacting space are stable during the simulation. On the other hand, a new H-bond ring in the C_9_(C=O···H–O···H–N) type is formed in front of the interacting space, and the structure of the tail region has the tendency to form another H-bond ring of the C_9_(C=O···H–O···H–N) type. The H-bonds in the C_9_(C=O···H–O···H–N) type can cause the distortion of the structure leading to the forming of a helix conformation. It can be concluded that the β-strand conformation included in the interacting space (M_3_ region) is transformed into a helix conformation due to the newly formed H-bonds.

It is a big challenge to understand the interactions in biomolecules at the atomic level with the surrounding water molecules because of their transient character and the inhomogeneity of H-bonding in liquid water [23]. Here, we have proposed a new kind of theoretical investigation on the interactions between peptide and explicit water molecules, and have been able to show the H-bond effects on the conformation transformation of the peptide as a first AIMD application.

## 4. Conclusions and Prospects

In this study, we presented the theoretical framework of the ELG-MD method and applied it to polyglycine (Gly)_14_ with 14 water molecules. Firstly, we tested ELG-MD over a short period of time and compared the results with those of conventional AIMD. We found from the comparison that the efficiency of ELG-MD is higher than the conventional AIMD method (about 31%). In addition, ELG-MD can easily be used to obtain the energy convergence of the system by treating only the local interacting space. The two methods produced similar trajectories with fixing of the same atoms. The advantage on efficiency and accuracy demonstrates the effectiveness of our ELG-MD method. Secondly, we performed 5ps (10,000 steps) ELG-MD simulation for this system. It was found from the simulation that the β-strand conformation in the (Gly)_14_ can be transformed into a random helix conformation by forming new H-bonds with the surrounding water molecules.

Although ELG-MD is not a full molecular dynamics, it can perform simulation in the active region which we are interested in, at the end of a polymer. In chain growth polymerization, the new monomer molecules add onto the active site on a growing polymer chain. The ELG-MD method is suitable for this kind of simulation. Many chemical reactions in biomolecular systems are similar to this process such as peptide chain elongation, the binding between inhibitor and enzyme *etc.* Although ELG-MD can only handle very simple quasi-one-dimensional systems at this stage, three-dimensional ELG-MD for more complicated real biomolecular systems is in progress [24]. 
